# Supplementary material for: Enhanced Electrocatalytic Water Oxidation of Ultrathin Porous Co3O4 Nanosheets by Physically Mixing with Au Nanoparticles
Source: Nanomaterials (Basel). 2022 Dec 11;12(24):4419. doi: 10.3390/nano12244419 (PMC9785958; doi:10.3390/nano12244419)
Supplement: Supplementary file 1 [file nanomaterials-12-04419-s001.zip › nanomaterials-2039993-supplementary.pdf]

nanomaterials

Supplementary Materials

Enhanced Electrocatalytic Water Oxidation of Ultrathin Porous Co_3O_4 Nanosheets by Physically Mixing with Au Nanoparticles

Changhe Hu [1,†], Dejuan Sun [2,†], Jie Liu [1], Qi Zhang [1], Xiao Li [1], Huhui Fu [1], M. Liu [1,2,*], Jiayue Xu [1], Guojian Jiang [1] and Yalin Lu [2,3,*]

1 School of Materials Science & Engineering, Shanghai Institute of Technology, Shanghai 201418, China
2 Department of Materials Science and Engineering, University of Science and Technology of China, Hefei 230026, China
3 Hefei National Laboratory for Physical Sciences at the Microscale, Hefei 230026, China
* Correspondence: liumin1106@ustc.edu.cn (M.L.); yllu@ustc.edu.cn (Y.L.)
† These authors contributed equally to this work.

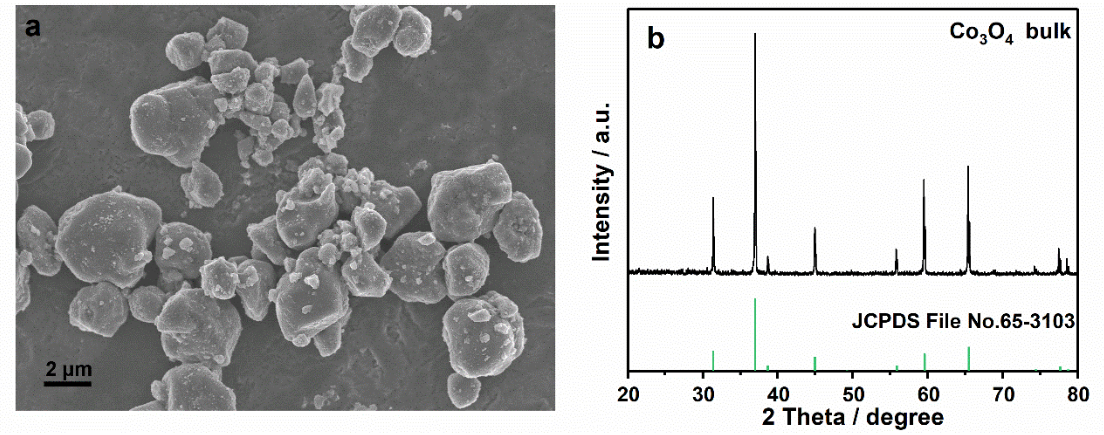

Figure S1. (a) SEM image and (b) XRD patterns of Co_3O_4 bulk.

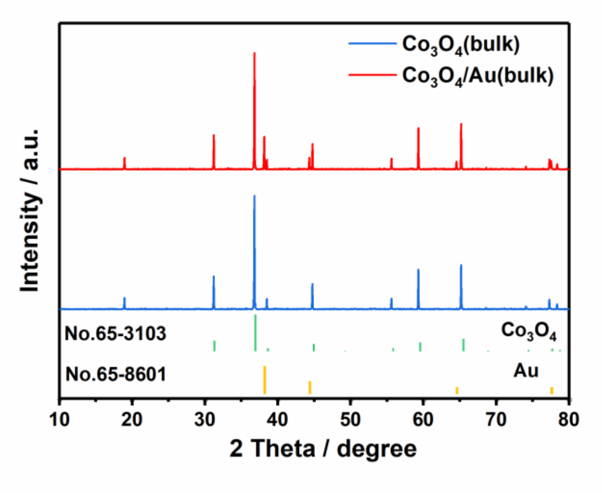

Figure S2. XRD patterns of Co_3O_4 bulk and Co_3O_4/Au bulk.

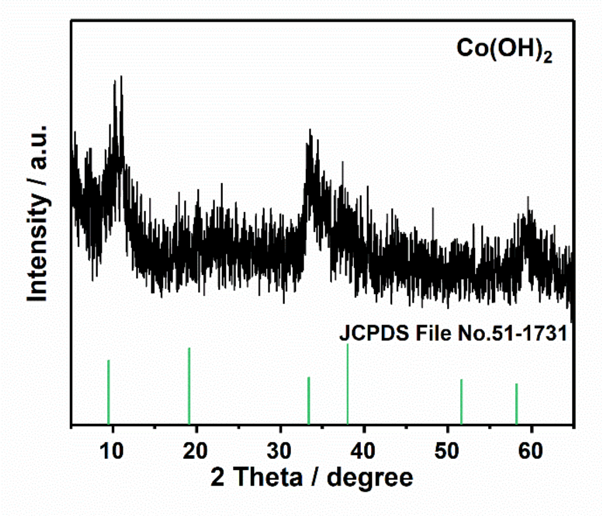

Figure S3. XRD pattern of $Co(OH)_2$ precursors.

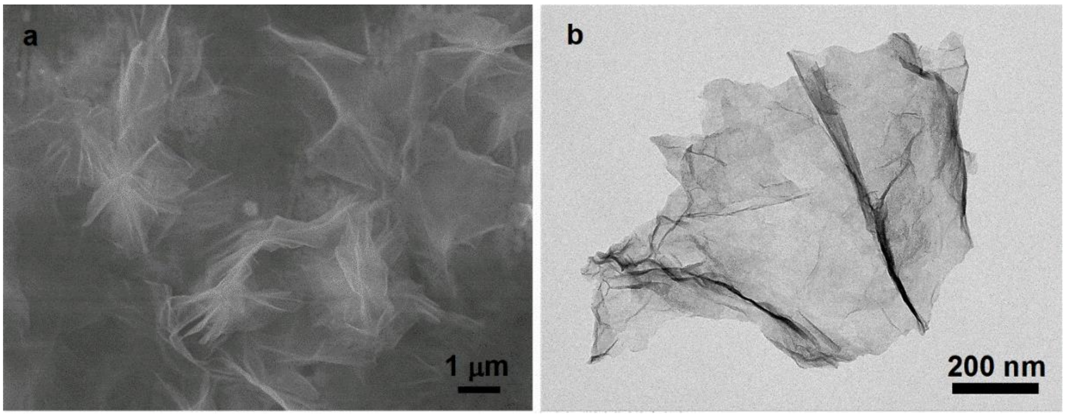

Figure S4. (a) SEM and (b) TEM images of $Co(OH)_2$ precursors.

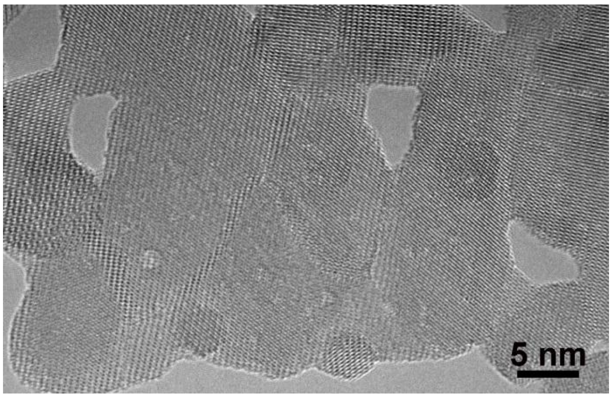

Figure S5. HRTEM image of the ultrathin porous Co_3O_4 nanosheets.

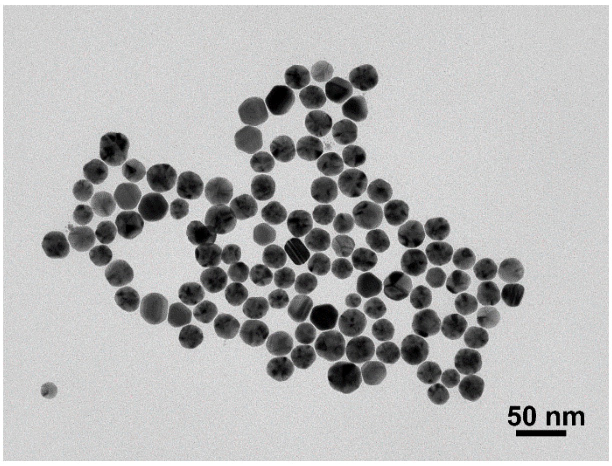

Figure S6. HRTEM image of Au nanoparticles.

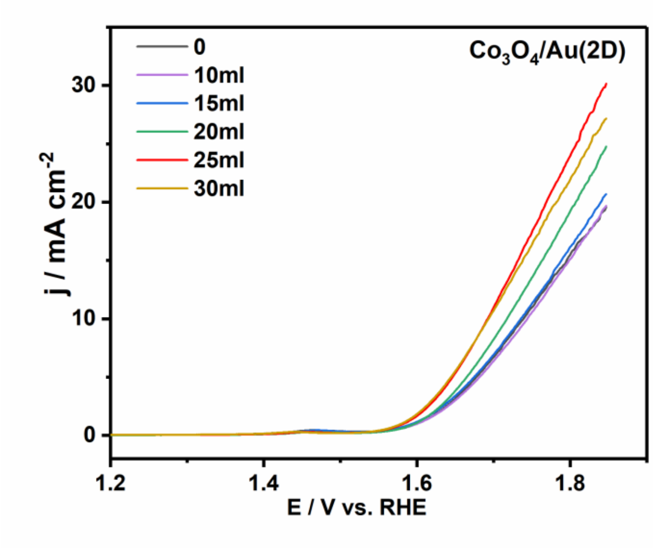

Figure S7. LSV curves of ultrathin porous Co_3O_4 nanosheets loading with different amount of Au nanoparticles.

The as–obtained Co_3O_4 nanosheets (15 mg) were dispersed in the solution with different amount of Au nanoparticles by stirring for 2 h. After freeze drying, the final products were obtained. With the increase of Au loading, the OER performance of nanocomposites is gradually enhanced, and reaches the best when 25 mL Au (9 wt%) is loaded, then declines as the further increase of Au loading due to the covering of active sites on the surface of Co_3O_4 nanosheets.

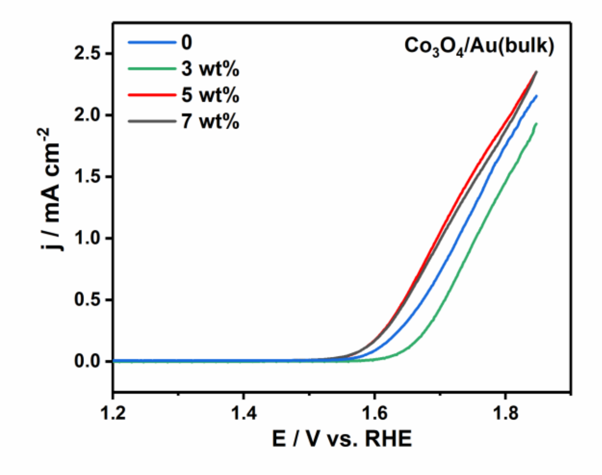

Figure S8. LSV curves of Co_3O_4/Au bulk loading with different amount of Au nanoparticles.

The Co_3O_4/Au bulk catalysts were prepared by an impregnation method. The Co_3O_4/Au bulk with various amount of Au loading were obtained by adjusting the amount of $HAuCl_4$.
